# Bibliometric Assessment of the Global Scientific Production of Nitazoxanide

**DOI:** 10.7759/cureus.1204

**Published:** 2017-05-01

**Authors:** Alfonso J. Rodriguez-Morales, Dayron F Martinez-Pulgarin, Marcela Muñoz-Urbano, Daniela Gómez-Suta, Jorge A Sánchez-Duque, Jorge E Machado-Alba

**Affiliations:** 1 Public Health and Infection Research Group, Faculty of Health Sciences, Universidad Tecnológica De Pereira, Pereira, Risaralda, Colombia; 2 Ophthalmology, Universidad Nacional de Colombia; 3 Grupo De Investigación En Farmacoepidemiologia Y Farmacovigilancia, Universidad Tecnológica De Pereira, Pereira, Risaralda, Colombia

**Keywords:** bibliometrics, chemotherapy, anthelminthics, antiprotozoal, antiparasitic, nitazoxanide, scientometrics

## Abstract

**Introduction:**

Nitazoxanide is a member of a new class of drug, thiazolides, and it was discovered in 1984 with antimicrobial activity effect against anaerobic bacteria, Hepatitis virus, protozoa, and helminths.

**Methods:**

A bibliometric study on four databases (1984-2016) – Medline, Scopus, LILACS, and SciELO – characterizing the global scientific production of nitazoxanide. We determined the quantity, quality (number of citations), and types of studies developed by each country, characterizing them by years, international cooperation, development, place of publication, authors (with its H-index), and groups with higher impact.

**Results:**

There were 512 articles in Medline – the higher scientific production is from the USA (19.71%), Switzerland (7.51%), and Mexico (7.27%). There were 1,440 articles in Scopus – from the USA (8.98%), Mexico (2.13%), and India (1.65%). There were 405 articles in LILACS – from Mexico (4.69%), the USA (4.2%), and Peru (2.47%). There were 47 articles in SciELO – from Brazil (34.04%), Venezuela (21.28%), and Colombia (14.89%). The H-index of nitazoxanide is 75 – the USA (26), Egypt (12), and Canada (10) were the countries contributing more with that.

**Conclusions:**

Nitazoxanide research has been highly important. Nevertheless, it is relatively limited when compared with other drugs. Its research has been led by the USA, as revealed in this bibliometric assessment. Although some developing countries, where it is used especially for protozoa and helminths, probably have its influence, and this explains the fact that Mexico and India, among others, are the top countries in the scientific production of this anti-infective agent. This bibliometric study evidenced a relatively low number of publications, however, it has been increased in recent years.

## Introduction

Nitazoxanide is a broad-spectrum antimicrobial drug. This antimicrobial inhibits the pyruvate ferredoxin oxidoreductase enzyme on the metabolism of some pathogenic microorganisms. Its spectrum includes mainly bacterial and parasitic (protozoa and helminths) species, as some viruses (Hepatitis B and C), which are etiologic agents of a wide range of diseases in territories of the developing world as Latin American and Asian countries, where it is frequently used [1].

This antimicrobial was initially classified as an antiparasitic agent due to its activity against *Taenia saginata* and *Hymenolepis nana*. After that, a higher spectrum was discussed, given the discovery of its antimicrobial activity against *Enterobacteriaceae*,* Trichomonas vaginalis*,* Entamoeba histolytica*, and *Clostridium difficile*. Nitazoxanide was considered an important therapeutic drug in metronidazole-resistant cases. In addition to that, the spectrum of nitazoxanide also includes activity against viral diseases such as hepatitis C. Currently, nitazoxanide is an important option to treat *Cryptosporidium *and *Giardia* infections, which are important etiologies of diarrhea in the pediatric population in developing countries, as well as in immunocompromised patients. Nitazoxanide is also an alternative therapy for *Clostridium difficile *diarrhea in inpatients [2-6]. In addition to the above uses, recent studies have found a promising anticancer effect of nitazoxanide, which could have a major impact on public health [7-8].

Nitazoxanide is a drug that preserves its spectrum and this has become as an important therapeutic tool, so it requires more investigation on potential future therapeutic effects [9]. Then, the objective of this study was to assess the scientific production of nitazoxanide in four international bibliographical databases.

## Materials and methods

A bibliometric study about the global scientific production on nitazoxanide was done. This assessment was performed in four important regional and international bibliographical databases, two of them in English and two in Spanish: Index Medicus/Medline/PubMed (www.pubmed.com) (English), analyzed through the GoPubMed® (http://gopubmed.com/web/gopubmed/) (January 1809-April 2017), Scopus (https://www.scopus.com/) from Elsevier (January 1959-April 2017) (English), SciELO (integrated) (http://scielo.org/php/index.php) (2004-2016) (Spanish) and LILACS (Latin American Literature on Health Sciences) (http://lilacs.bvsalud.org/es/) (1980-2016) (Spanish). All the regions of the world, as well as registered countries in the databases, were searched. As these databases have different literature coverage, results are presented per *database*, due to the fact that it is not technically possible to integrate them, as they are not of the same quality too, among other limitations.

This research strategy used the following keywords (MeSH, Medical Subject Headings): "Nitazoxanide (nitazoxanide)" AND "Argentina", "Nitazoxanide (nitazoxanide)" AND "Zimbabwe" and in the same way as the rest of countries. We determined the quantity, quality (express as the number of citations, more of them more relative quality), and types of studies performed by each country, characterizing them for years, international cooperation (IC), country of publication (COP), citations and H-index, authors and groups with the highest contribution.

Data was tabulated and analyzed in Excel 365® for Windows 8®, summarizing quantitative variables with means and standard deviations (±DE) and qualitative with proportions.

## Results

### Medline

**Figure 1:**
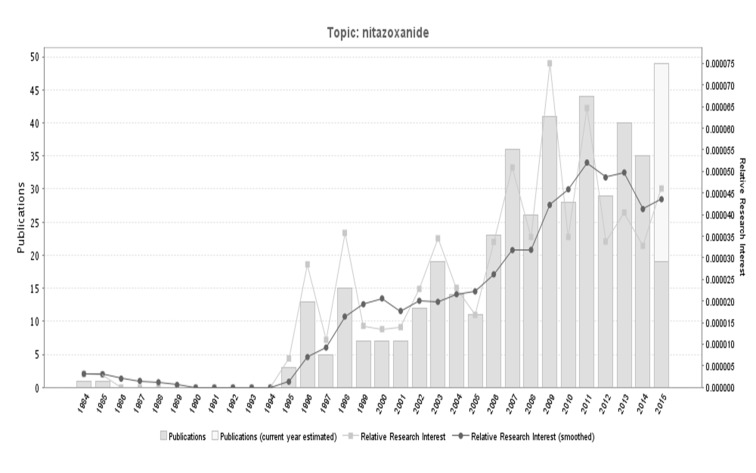
Evolution over time of publication on nitazoxanide in Medline

In Medline, we found 512 articles published and indexed in the study period, with a clear trend to increase their research in the last decade (Figure 1).

**Figure 2:**
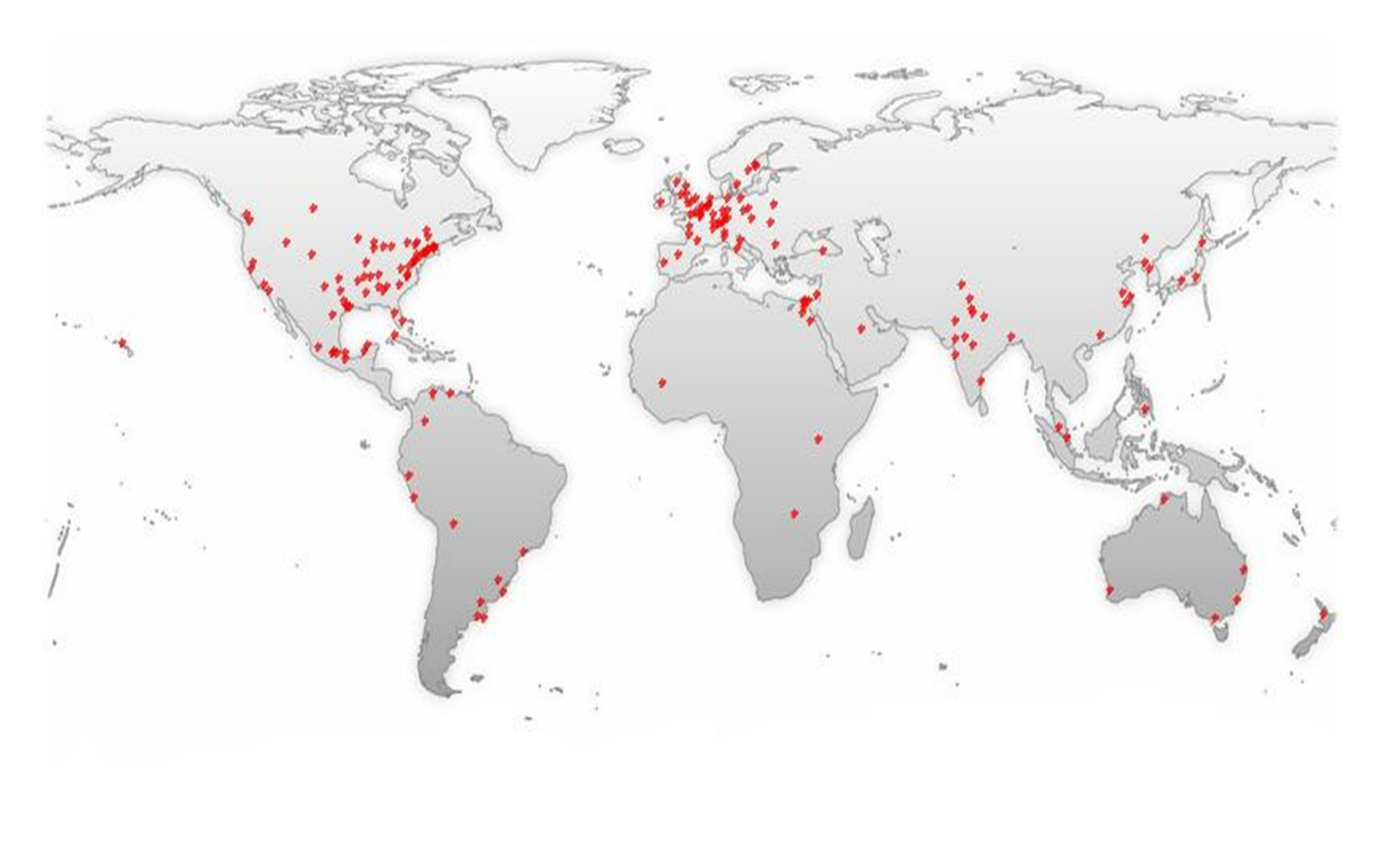
International scientific production by place related to nitazoxanide in Medline

Analyzing the results by world regions, Europe leads scientific research on nitazoxanide, followed by North America with the largest scientific production (Figure 2).

**Figure 3:**
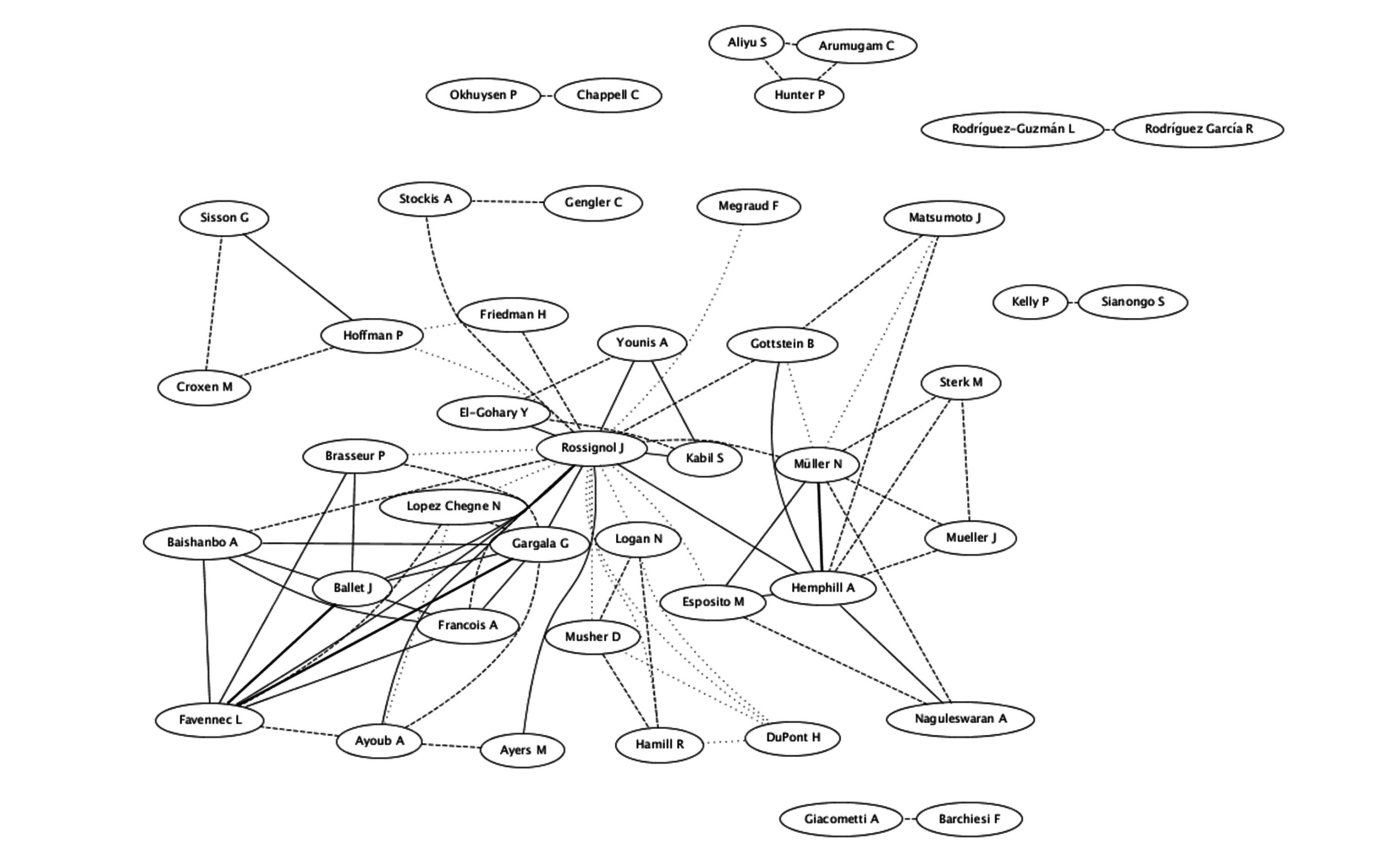
International cooperation on scientific production of nitazoxanide in Medline

Regarding international cooperation, the group of Dr. Jean-François Rossignol, of the USA, who developed the drug, is the one which has had major interactions with other authors and research groups both nationally and internationally, with more than 150 co-authors, an H-index of 33 and 2,910 citations (Figure 3).

**Figure 4:**
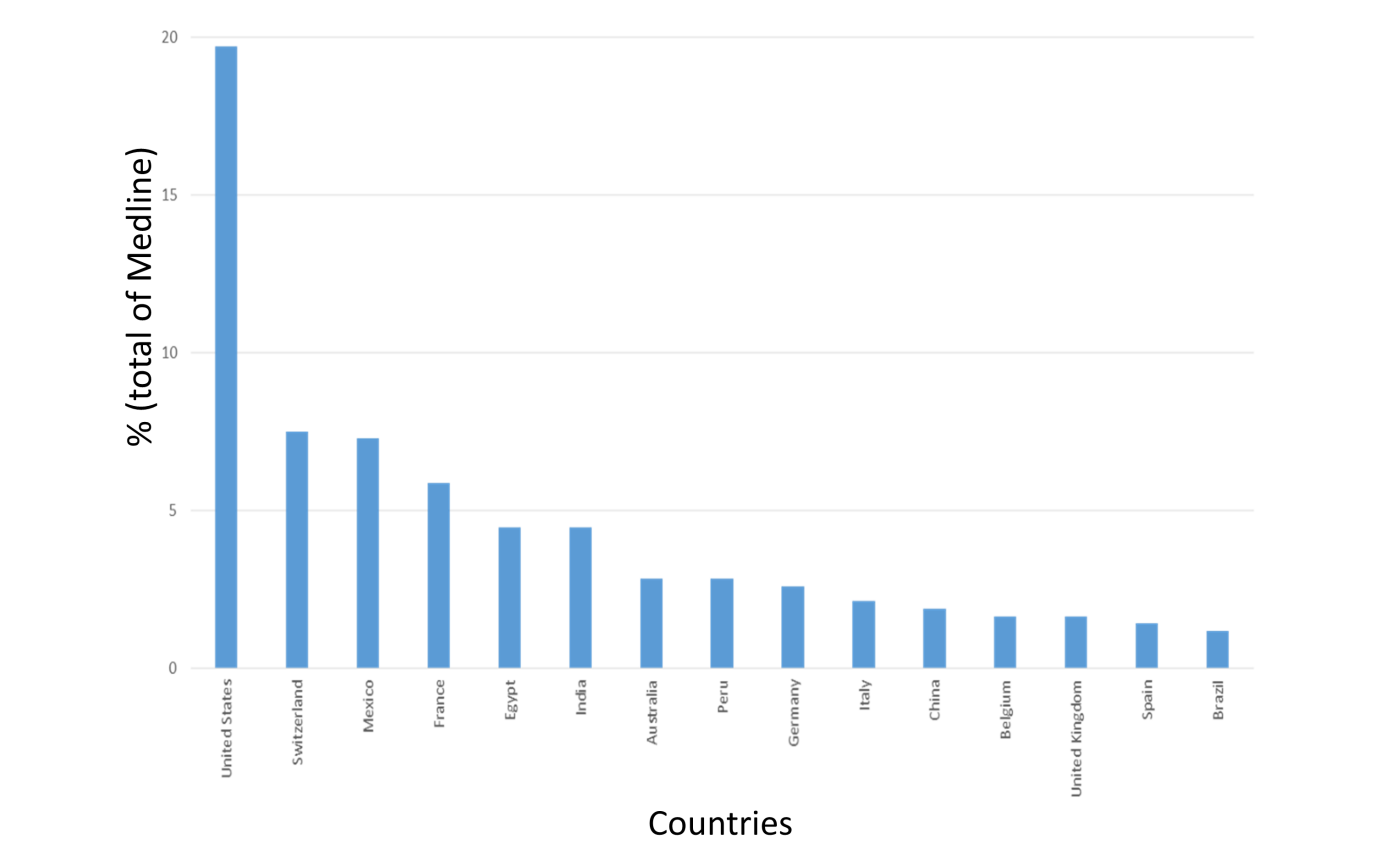
Countries with the highest scientific production of nitazoxanide in Medline

The cooperation of that group extends to countries such as France, UK, Egypt, Switzerland, Belgium, Italy, China, Peru, Canada, Germany, Japan, Mali, Mexico, and Pakistan, among others (Figure 3). In these countries, it has concentrated the largest scientific production, led by the USA with 19.71%, followed by Switzerland with 7.51% and Mexico with 7.27%, among other (Figure 4).

### Scopus

**Figure 5:**
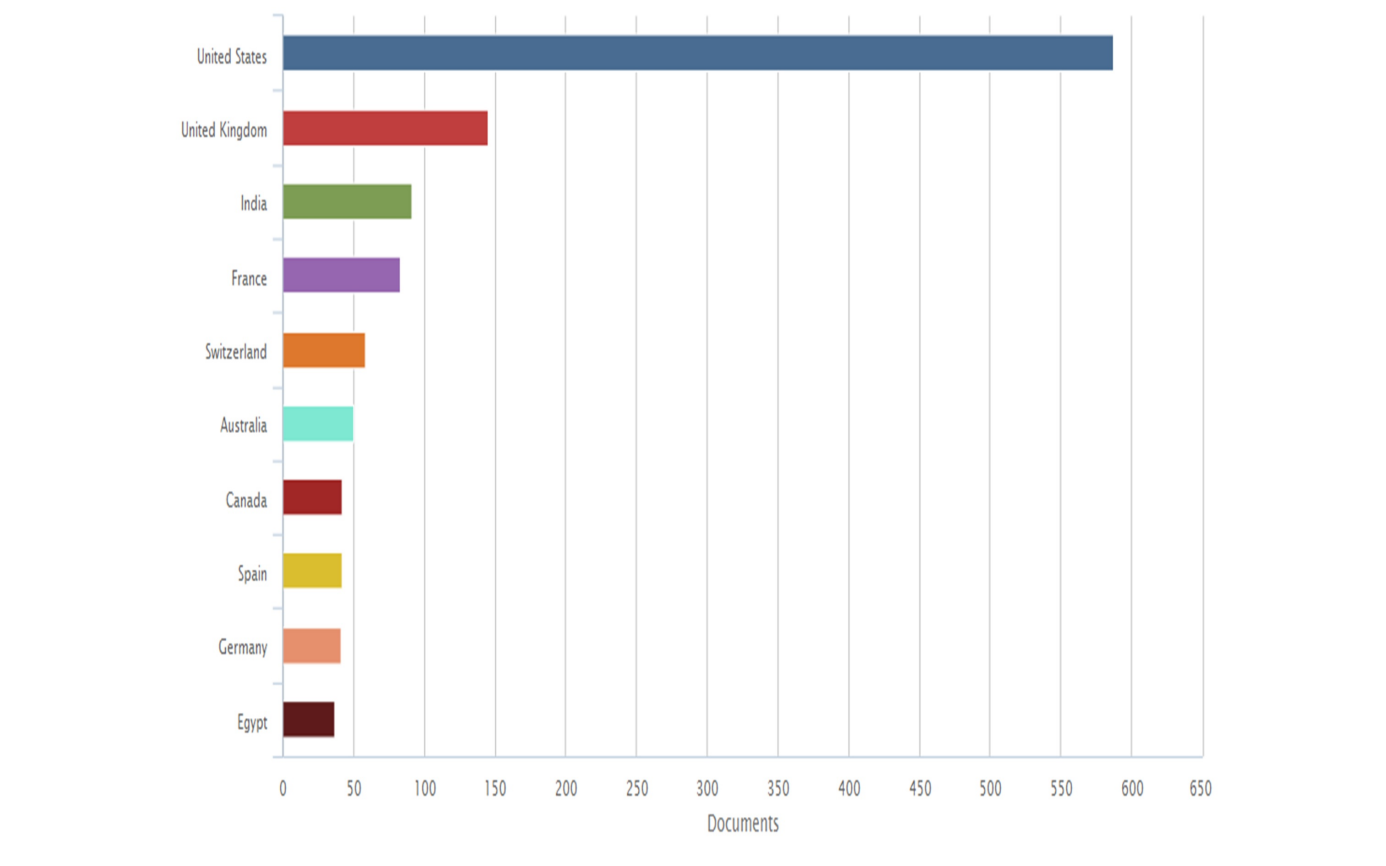
International scientific production by place related to nitazoxanide in Scopus

In Scopus, we found 1,440 articles published, 8.98% of USA, 2.13% of Mexico, and 1.65% of India, among others (Figure 5).

**Figure 6:**
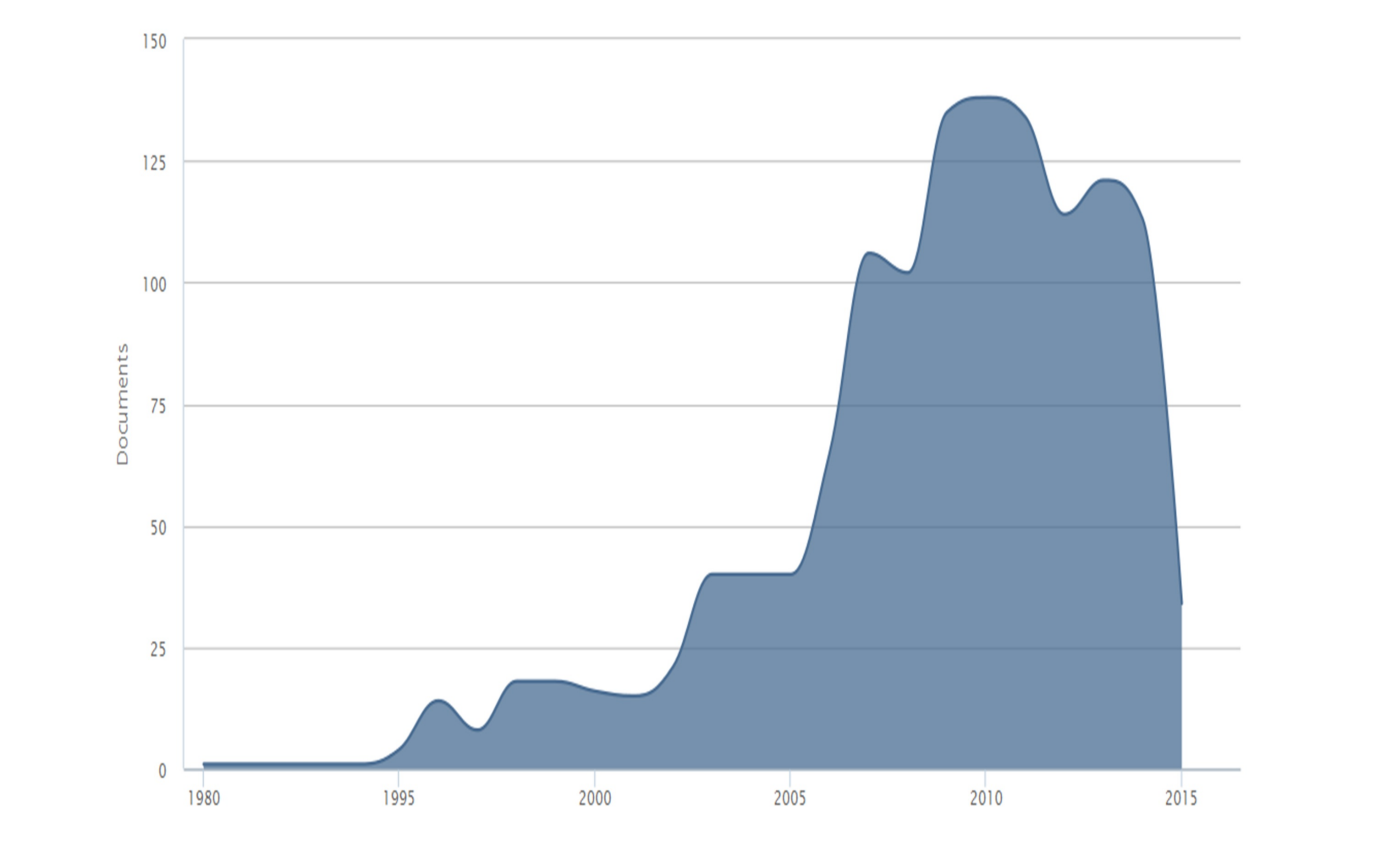
Evolution over time of publication on nitazoxanide in Scopus

Whereas in this database, there is a clear trend to increase the scientific production over the last decade (Figure 6).

**Figure 7:**
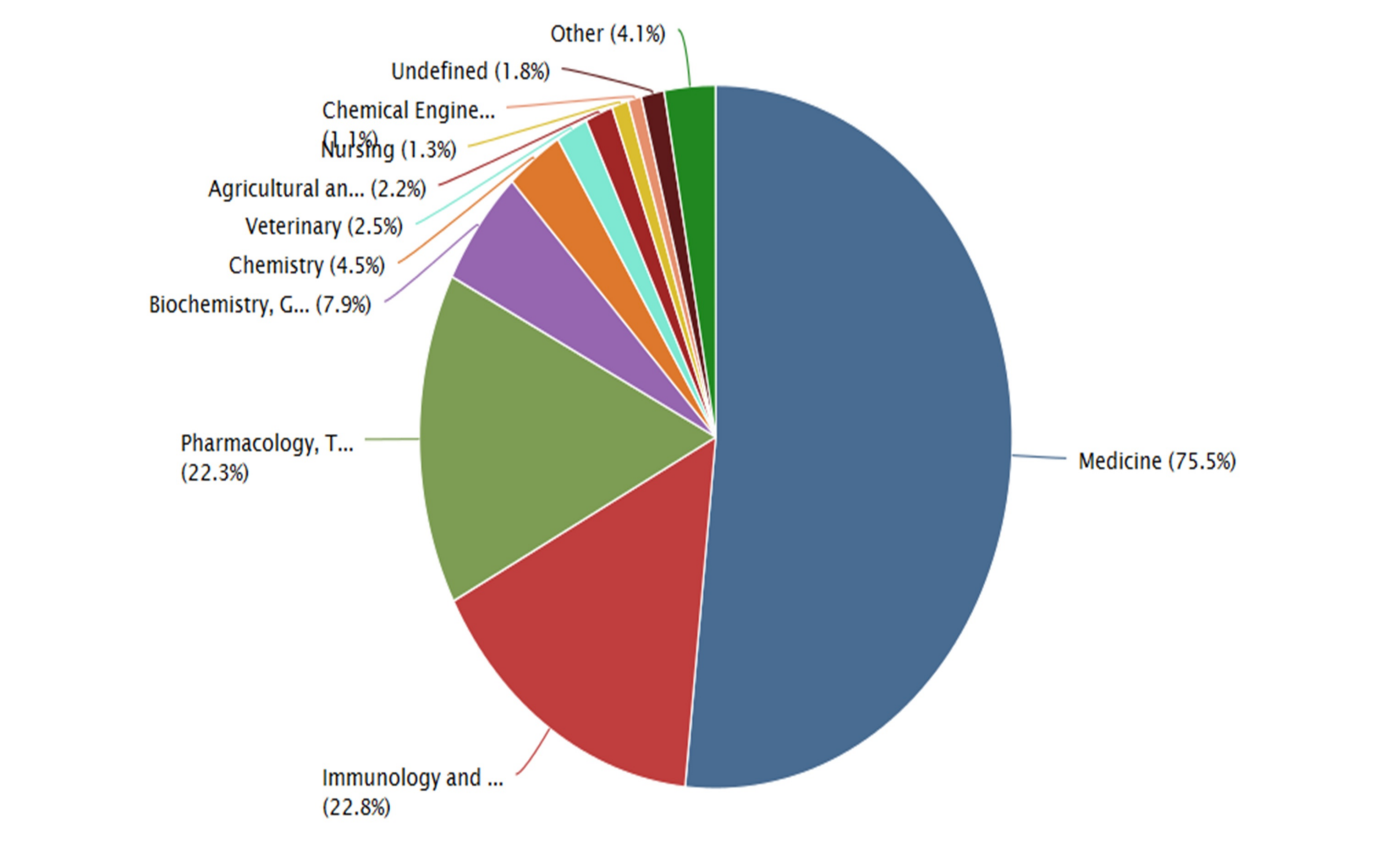
Areas of scientific research on nitazoxanide in Scopus

In Scopus, it shows that the *area of knowledge,* which belongs to the scientific production of Nitazoxanide, is dominated by Medicine (75.5%), followed by immunology and microbiology (22.8%), and pharmacology, toxicology and pharmaceuticals (22.3%) (Figure 7).

**Figure 8:**
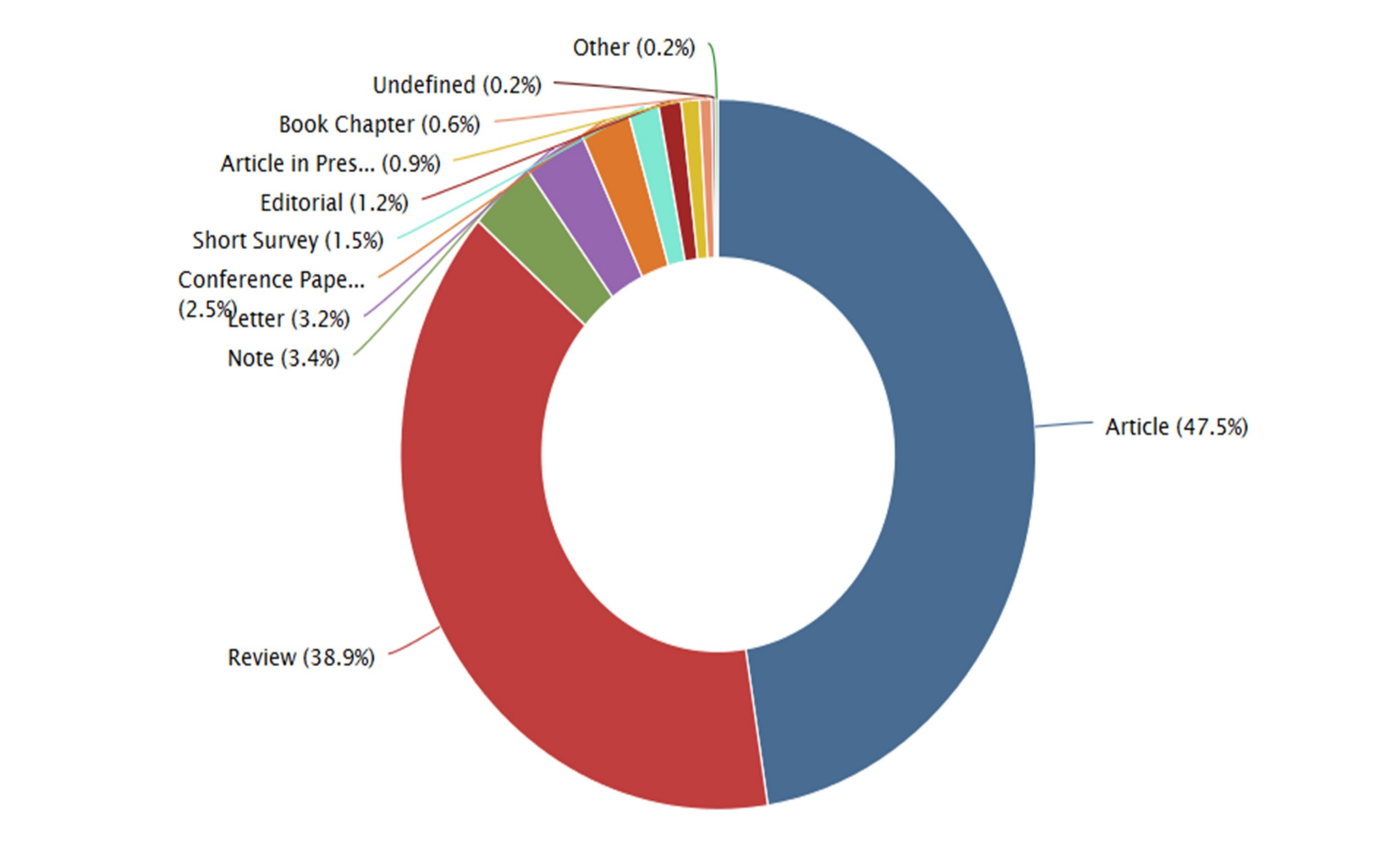
Types of article published on nitazoxanide in journals indexed in Scopus

Regarding the types of contributions, it was observed that the largest share corresponded with original papers (article) (47.5%), followed by review articles (38.9%), among other contributions (Figure 8).

**Figure 9:**
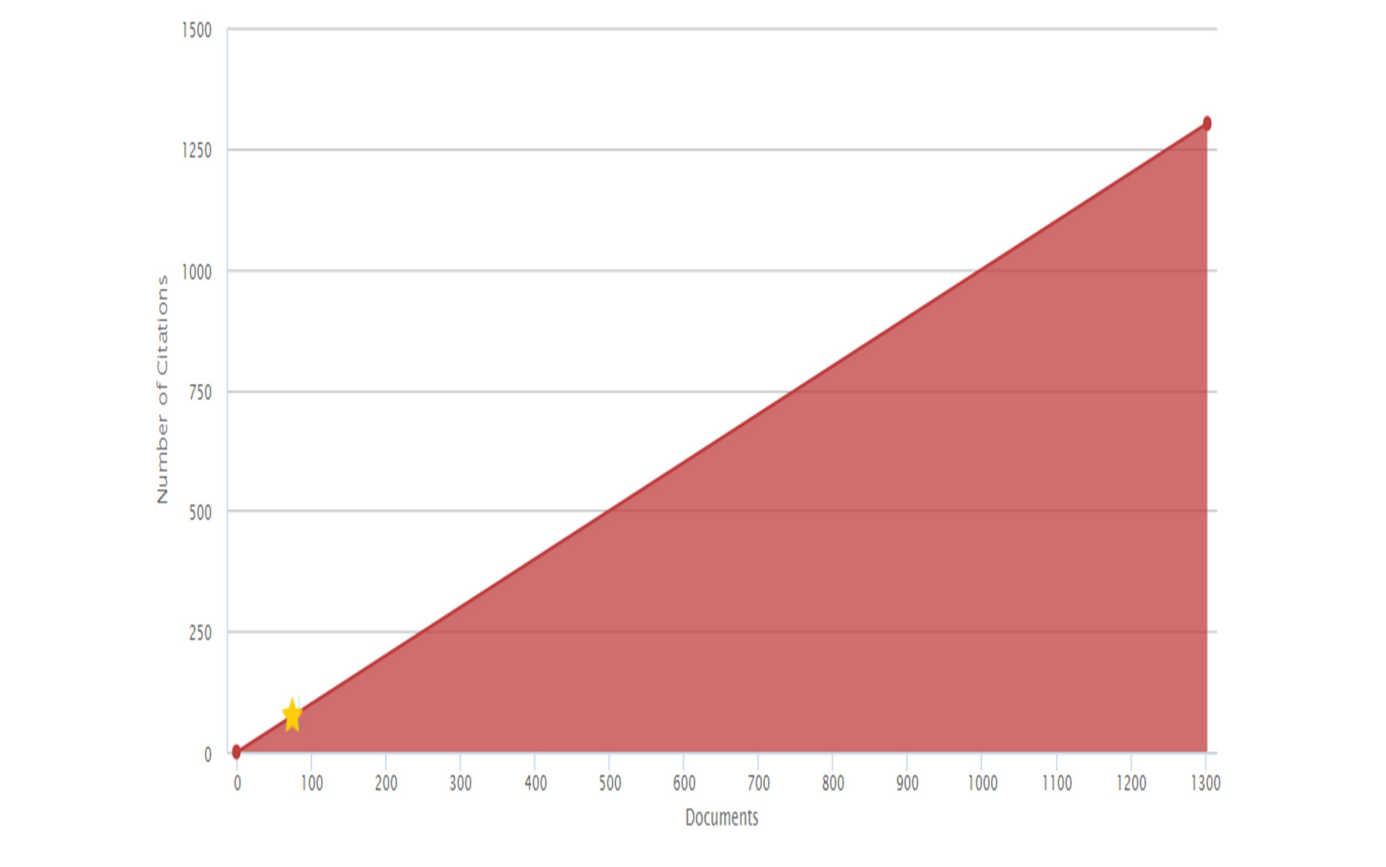
H-index of nitazoxanide

Concerning the quality of research, assessed as citations, studies about nitazoxanide have received 25,597 citations, for a general H-Index of 75 (Figure 9).

Being the highest H-index by a country retrieved for the USA (H = 26), followed by Egypt (H = 12) and Canada (H = 10), which are the countries with the highest number of citations, 2305, 677 and 630, respectively.

### SciELO

**Figure 10:**
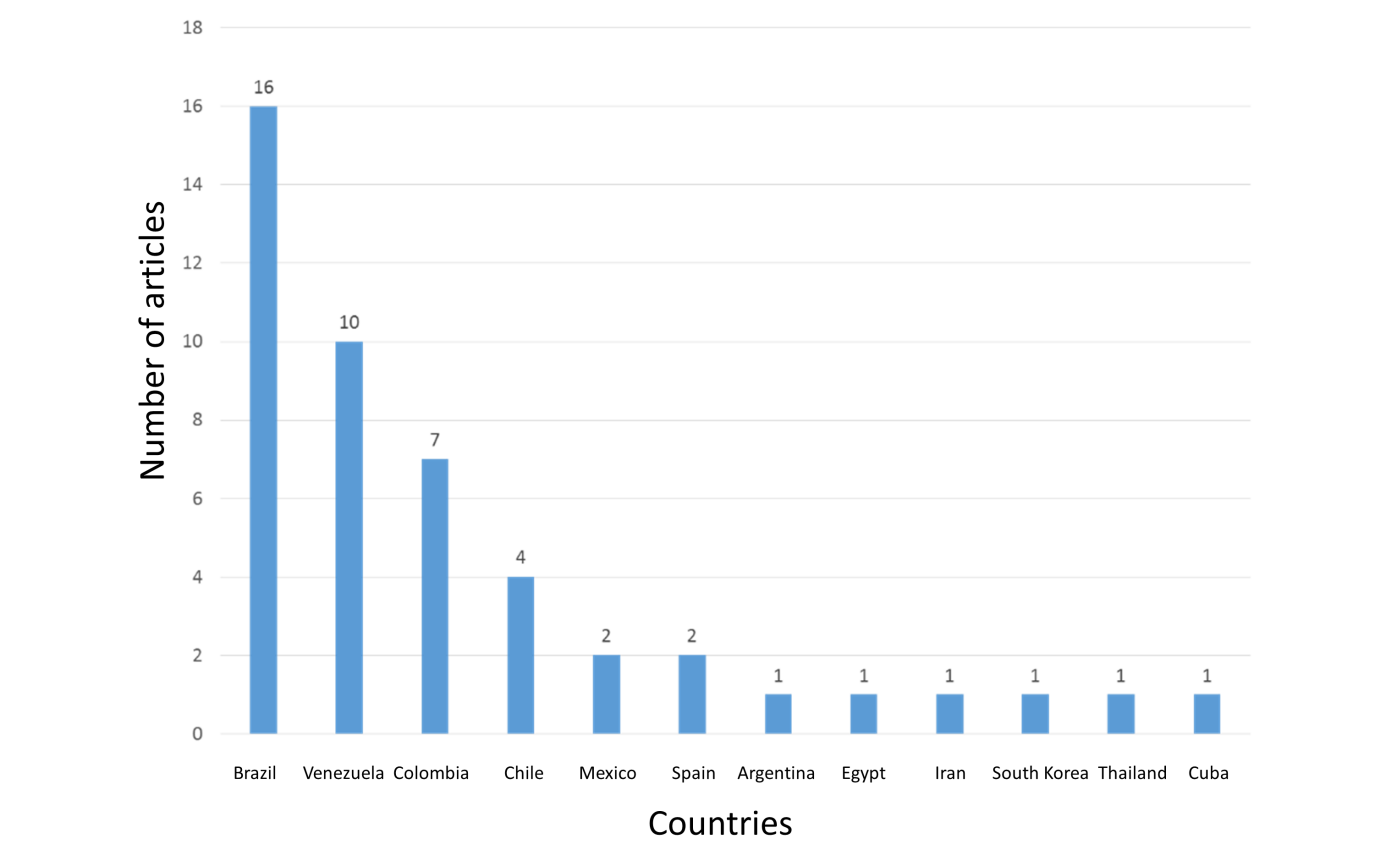
Countries with the highest scientific production of nitazoxanide in SciELO

In the regional Scielo database, only 47 articles were found, of which 34.04% are from Brazil, 21.28% from Venezuela, and 14.89% from Colombia, among others (Figure 10).

### Lilacs

**Figure 11:**
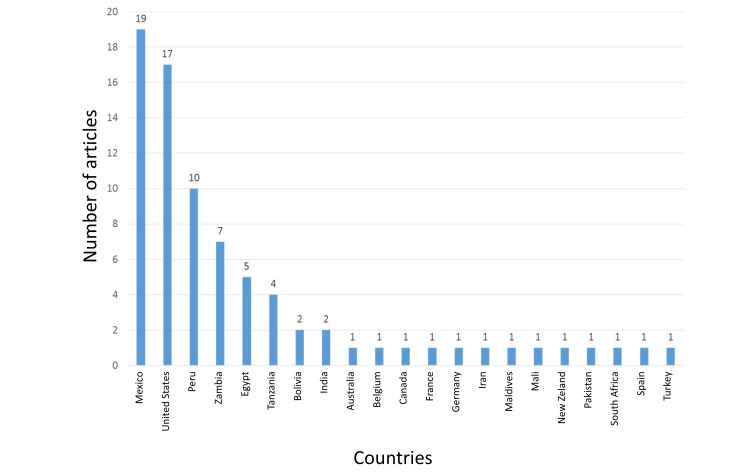
Countries with the highest scientific production of nitazoxanide in Lilacs

Finally, in LILACS, we found 405 items, of which 4.69% are from Mexico, 4.2% from the USA, and 2.47% from Peru, among others (Figure 11).

## Discussion

Nitazoxanide has been an important antimicrobial drug. This has generated significant research in the world. In this study, it was demonstrated the leadership of the USA about nitazoxanide global research (10% of the global contributions), which doubled Switzerland, the second in research production of it. Nevertheless, it is clear that there is still a limited scientific production on this drug. However, after the 1980s, there was an exponential rising in the production, especially in the last 10 years, which counts for the largest scientific production of the topic (Figures 1, 5). Most of the research is represented by original articles (47.5%), supporting the fact that the innovation of this medication is increasing everyday (Figure 8). Due to its public health implications, the use of this drug is of particular interest for treatment, control and potentially prevention of tropical and parasitic diseases which occur due to protozoa and helminths.

The above-mentioned information could be explained by the new findings of the mechanisms of action and uses, like its use against plenty of viruses such as rotavirus, hepatitis B (HBV) and C (HCV), yellow fever, dengue, influenza [1, 4, 10-12]. Countries such as the USA, Switzerland, France, United Kingdom, Mexico, and Brazil have shown the highest rates of contributions on the subject (Figures 6, 10-11). In the case of Mexico and Brazil, parasitic diseases due to protozoa and helminths are still highly prevalent, especially in rural areas. Then its use and the research of its efficacy and safety would be expected, also considering that both countries are the main contributors of Latin America's biomedical and non-biomedical research. Then, currently and probably in the future, nitazoxanide research would be of high importance in public health.

One of the possible explanations for the significant increase in research about this drug in recent years is due to an augmented resistance to antibiotics, thereby serving as an alternative medication for a high impact public health issue as has been considered recently by World Health Organization (WHO) [9, 13-14].

As occurred in multiple other topics, related to tropical diseases [15-19], affecting significantly developing countries, research on them is led by developed countries. This situation is also seen with research on nitazoxanide. For example, as expected, in Latin America, Brazil led research on nitazoxanide, as well as on other topics related to tropical diseases (Figure 2). In this region, other countries, such as Venezuela, Colombia, and Chile, also contributed on research, for emerging topics related to its use (e.g., use in toxocariasis) [20] (Figure 10). Nevertheless, its clinical use for toxocariasis is still to be better defined, then requiring more research, especially in these countries.

Besides that, leading research groups on this drug are located in developed countries, where our analysis showed that Dr. Jean-François Rossignol, from the USA, is a leading author, but interestingly this researcher is a piece integrating over a dozen of institutions in different parts of the world (Figure 3) in a large cooperation network.

Potential limitations of the study are related to the database used to retrieve articles. Further studies should include Science Citation Index, which was not included because of the costs of subscription for the institution (not affordable). Included databases do not represent all scientific and biomedical journals published; in fact, many articles of importance may appear in journals other than those indexed in used databases or may not be indexed as the case of contributions made to scientific conferences and meetings. However, the recognized quality of the publications included in these databases and their coverage means that the documents selected constitute a more than representative sample of the international research on nitazoxanide [15-19]. In the near future, it would be interesting also to perform studies on the prescribing patterns and costs of this drug use by countries, as has been now commonly reported in Colombia for other drugs [21].

Bibliometric studies are currently important to define the status of research on any topic, particularly of health, of diseases as well as, in this case, of drugs that have been developed and with clinically significant implications, as is the case of nitazoxanide.

## Conclusions

Despite its methodological limitations, this study represents the first effort, based on the literature review, to explore the development and research productivity on nitazoxanide over time and has pointed out interesting characteristics of nitazoxanide literature. First, the concentration of papers over journals is disseminating the research results, and second, the research in this field is growing and possibly will continue in the years to come. From these findings, it is clear that countries such as the USA lead the research on this drug, but it is evident that there is a need to increase this drug in developing countries, where its use is highly important due to its anti-infective coverage, especially in tropical parasitic diseases.

Bibliometric studies in different areas of medicine are of great importance, not only to map research needs in a particular subject but also, as shown here, to throw an accurate view of scientific production over time and their impact in future years, for example, in research on drugs and in particular on anti-infective agents. These results would also be important for the analyses of funding research agencies as well as to academic institutions with interest in the topic. We recommend finally to perform periodically this type of assessment to keep track trends of research in this and other anti-infective drugs.
